# Expression of the circular RNAs in astaxanthin promotes cholesterol efflux from THP-1 cells based on RNA-seq

**DOI:** 10.1186/s12263-021-00693-5

**Published:** 2021-08-28

**Authors:** Jie Liu, Yue Wei, Yong Lin, Peiwen Zhang, Zhexiao Zhang, Hairong Huang, Hongfu Wu, Tangbin Zou

**Affiliations:** 1 Department of Ultrasound, Shunde Women and Children’s Hospital of Guangdong Medical University (Maternity & Child Healthcare Hospital of Shunde Foshan), 528300 Foshan, China; 2 Dongguan Key Laboratory of Environmental Medicine, School of Public Health, Guangdong Medical University, Dongguan, 523808 China; 3 Department of Surgery, the Third Affiliated Hospital of Guangdong Medical University (Longjiang Hospital of Shunde District), 528318 Foshan, China; 4 Key Laboratory of Stem Cell and Regenerative Tissue Engineering, Guangdong Medical University, Dongguan, 523808 China

**Keywords:** Astaxanthin, Cholesterol efflux, Circular RNAs, RNA sequencing

## Abstract

**Background:**

It is reported that circular RNAs (circRNAs) play a key role in atherosclerosis (AS). Foam cell formation, which is the main feature of AS, can be significantly inhibited by cholesterol efflux.

**Methods:**

We established a model of astaxanthin (AST) promoting cholesterol efflux from macrophages through oil red O staining, real-time quantitative PCR (qRT-PCR), and western blot and used RNA sequencing to detect the expression of circRNAs in AST-treated and untreated THP-1 cells. Finally, siRNA transfection screened out circRNAs that were significantly differentially expressed. The data analysis was performed by Student’s *t* test and *P* < 0.05 was considered statistically significant.

**Results:**

In the model of AST promoting cholesterol efflux from THP-1 cells, there were a total of 7276 circRNAs differentially expressed, among which the top 25 upregulated and the top 25 downregulated circRNAs were selected based on the log_2_ (fold change). GO analysis showed that differential expression of circRNAs in biological process (2066/3098; 66.69%), molecular function (543/3098; 17.53%), and cellular component (489/3098; 15.78%). Based on KEGG analysis, RNA transport was the most enriched pathway. Finally, we obtained 3 significantly upregulated circRNAs by siRNA transfection and qRT-PCR.

**Conclusions:**

The 3 differentially expressed circRNAs may play an important role in the process of AST promoting cholesterol efflux and may be used as biomarkers to prevent AS.

**Supplementary Information:**

The online version contains supplementary material available at 10.1186/s12263-021-00693-5.

## Introduction

Atherosclerosis (AS) cardiovascular disease is one of the main causes of morbidity and mortality in the world, and it has become an important disease threatening public health [1]. Heart disease (the most common cause is atherosclerotic disease of the coronary arteries) and stroke are the two leading causes of death in the world, and it has gradually become a common disease that seriously endangers people’s health [2]. One of the characteristics of atherosclerosis is the accumulation of lipids in the arterial wall. The main risk factors include high levels of low-density lipoprotein cholesterol (LDL-C) and low levels of high-density lipoprotein cholesterol (HDL-C) [3, 4]. The function of LDL is to transport endogenous cholesterol and lipids from the liver to the periphery; while the function of HDL is to reverse cholesterol transport (RCT), and to transport lipids from the periphery to the liver for catabolism [5]. The process of transporting excess cholesterol from the surrounding tissues such as macrophages to the liver through HDL for metabolism and excretion and finally excretion of feces, that is, RCT, can effectively inhibit the occurrence of AS [5–7]. Foam cell formation is an important part of AS, and cholesterol efflux, as the initial and key step of RCT, can effectively inhibit the formation of foam cells, thereby preventing the occurrence of AS [7, 8]. Therefore, promoting cholesterol efflux and enhancing the ability of RCT are important strategies to reduce the risk of AS. Cholesterol transporters such as ATP-binding cassette transporter A1 (ABCA1), G1 (ABCG1) and scavenger receptor class B type I (SR-BI) are responsible for regulating the cholesterol output of macrophages and participates in the RCT [4, 8]. Their increased expression can promote RCT, reduce AS, and play a key role in preventing the accumulation of cholesterol in macrophages [9, 10].

Astaxanthin (AST), as a non-vitamin A pro-carotenoid with the strongest known antioxidant activity, can exert its powerful antioxidant activity by quenching singlet oxygen and scavenging free radicals [11]. Studies have shown that AST can reduce the content of total cholesterol and LDL-C, and significantly increase the level of HDL-C [12]. Clinical studies have found that AST can also inhibit the oxidized low-density lipoprotein (ox-LDL) and apolipoprotein (apoA-I) and increase the levels of HDL-C and adiponectin, playing an important role in preventing the development of AS [11, 13, 14]. Studies have found that AST can increase the expression of ABCA1/G1, thereby enhancing the apoA-I/HDL-mediated cholesterol efflux of macrophages, thereby preventing AS [15].

Circular RNA (circRNA) is a covalently closed circular RNA molecule produced by reverse splicing of pre-mRNA (pre-mRNA) and is a special non-coding endogenous RNA [16]. CircRNAs have a variety of biological functions such as acting as a “microRNA sponge,” regulating protein interactions, acting as a protein sponge or scaffold, and participating in ribosomal RNA processing and translation processes [17, 18]. At present, the research on the mechanism of circRNAs in disease is more inclined to act as a miRNA sponge. It may be used as a biomarker or therapeutic target for AS and cancer and protects the development of AS [19]. Studies have found that circ-SATB2 and circCHFR upregulate the expression of related target genes by sponging miR-939 and miR-370, respectively; to regulate the proliferation and differentiation of vascular smooth muscle cells, and play a vital role in atherosclerosis in cardiovascular and cerebrovascular diseases [20, 21]; circRNA-0044073 is upregulated in AS by targeting miR-107 and activating the JAK/STAT signaling pathway, which may provide a target for new anti-atherosclerotic treatment strategies [22].

However, the molecular mechanism of circRNAs in promoting cholesterol efflux from macrophages is still rarely reported. Therefore, this article mainly studies the differentially expressed circRNAs and their targeted miRNA in the process of AST promoting cholesterol efflux, laying a foundation for in-depth exploration of the specific mechanism of circRNAs in this process.

## Results

### AST inhibited foaming of macrophages and promoted cholesterol efflux

**Fig. 1:**
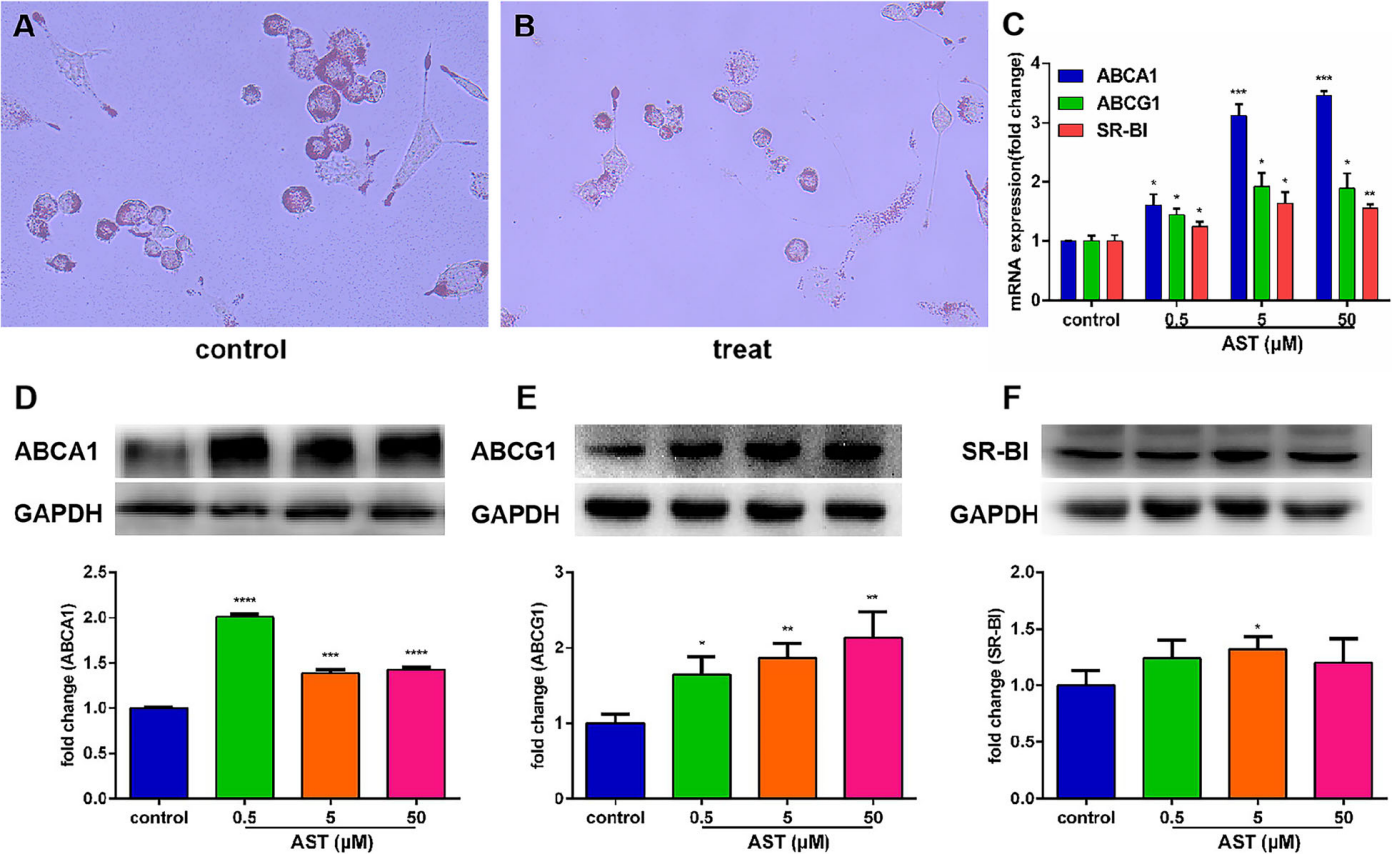
AST inhibited foaming of macrophages and promoted cholesterol efflux. Oil red O staining showed that compared with the control group (**A**), the red area in the cells of the treat group (**B**) was significantly reduced after AST treatment. qRT-PCR (**C**) and western blot (**D**–**F**) experiments to detect the effects of different concentrations of AST on the expression of ABCA1, ABCG1, and SR-BI genes and protein levels after intervention. *P < 0.05 vs. control. **P < 0.01 vs. control. ***P < 0.001 vs. control. ****P < 0.0001 vs. control

To evaluate the inhibitory effect of AST on macrophage foam cell formation, THP-1 cells were loaded with 50 μg/ml ox-LDL after PMA (Phorbol-12-myristate-13-acetate)-induced adherence as cell culture models. The result of Oil Red O staining showed that compared with the control group (Fig. 1A), the red part of the cells in the treat group (Fig. 1B) was significantly reduced, indicating that the AST significantly inhibited ox-LDL-induced macrophage foam cell formation. At the same time, treatments with different concentrations (0, 0.5, 5, 50 μM) of AST were set up, and real-time quantitative PCR (qRT-PCR) and western blot experiments were used to detect the expression of cholesterol efflux-related genes and proteins ABCA1, ABCG1, and SR-BI. The results showed that the expression of ABCA1, ABCG1, and SR-BI genes was significantly increased after AST treatment compared with the control group (Fig. 1C). In addition, the expression of ABCA1, ABCG1, SR-BI protein also increased to varying degrees (Fig. 1D–F). It can be seen that different concentrations of AST can gradually upregulate the expression of ABCA1, ABCG1, SR-BI, and the effect is better at 5 μΜ concentration.

### Differential expression of circRNAs

We drew the volcano plot and the scatter plot to visually evaluate the difference in circRNAs expression between the treat and control groups (Supplementary Fig. S1A-B). The values spotted in the *X* and *Y* axes represent the average of normalized signals of the two groups of samples (log_2_ scaled). The circRNAs above the top red line and below the bottom green line indicate more than 2 fold change in circRNAs between the two groups. The heat map indicated the expression profiles of all circRNAs (Supplementary Fig. S1C). RNA sequencing (RNA-seq) detected significantly differential expression of 7276 circRNAs between the treat cells and control cells (|log_2_ (fold change)| > 2), of which 3116 circRNAs were upregulated and 4160 circRNAs were downregulated.

### Functional enrichment analysis

Gene ontology (GO)-based enrichment analysis was carried out to evaluate the major biological functions of differentially expressed circRNA-miRNA-mRNAs network that are further classified into three main categories such as, biological process (2066/3098; 66.69%), molecular function (543/3098; 17.53%) and cellular component (489/3098; 15.78%) (Supplementary Fig. S2A). The top 10 of each subcategory along with the analysis of all circRNA-miRNA-mRNA networks between the treat and control groups are shown in the significantly enriched GO dendrogram (Supplementary Fig. S2B). The ten most-enriched GO biological processes were mainly associated with metabolic processes, including cellular metabolic process, primary metabolic process, organic substance metabolic process, nitrogen compound metabolic process, and organic substance. Of note, catabolic processes were also significantly enriched. The ten most-enriched GO cellular components were intracellular part, organelle part, cytoplasm, nuclear, and cytosolic, and the ten most-enriched GO molecular functions included nucleoside binding, small molecule binding, protein binding, RNA binding, and ATP binding.

KEGG (Kyoto Encyclopedia of Genes and Genomes) pathway analysis revealed 20 substantially enriched pathways, many of which are relevant to the transport and translation. RNA transport was the most enriched pathway, followed by endocytosis, cell cycle, and lysosome signaling pathway in treat and control comparisons (Supplementary Fig. S3).

### Confirmation of differentially expressed circRNAs by qRT-PCR

**Fig. 2:**
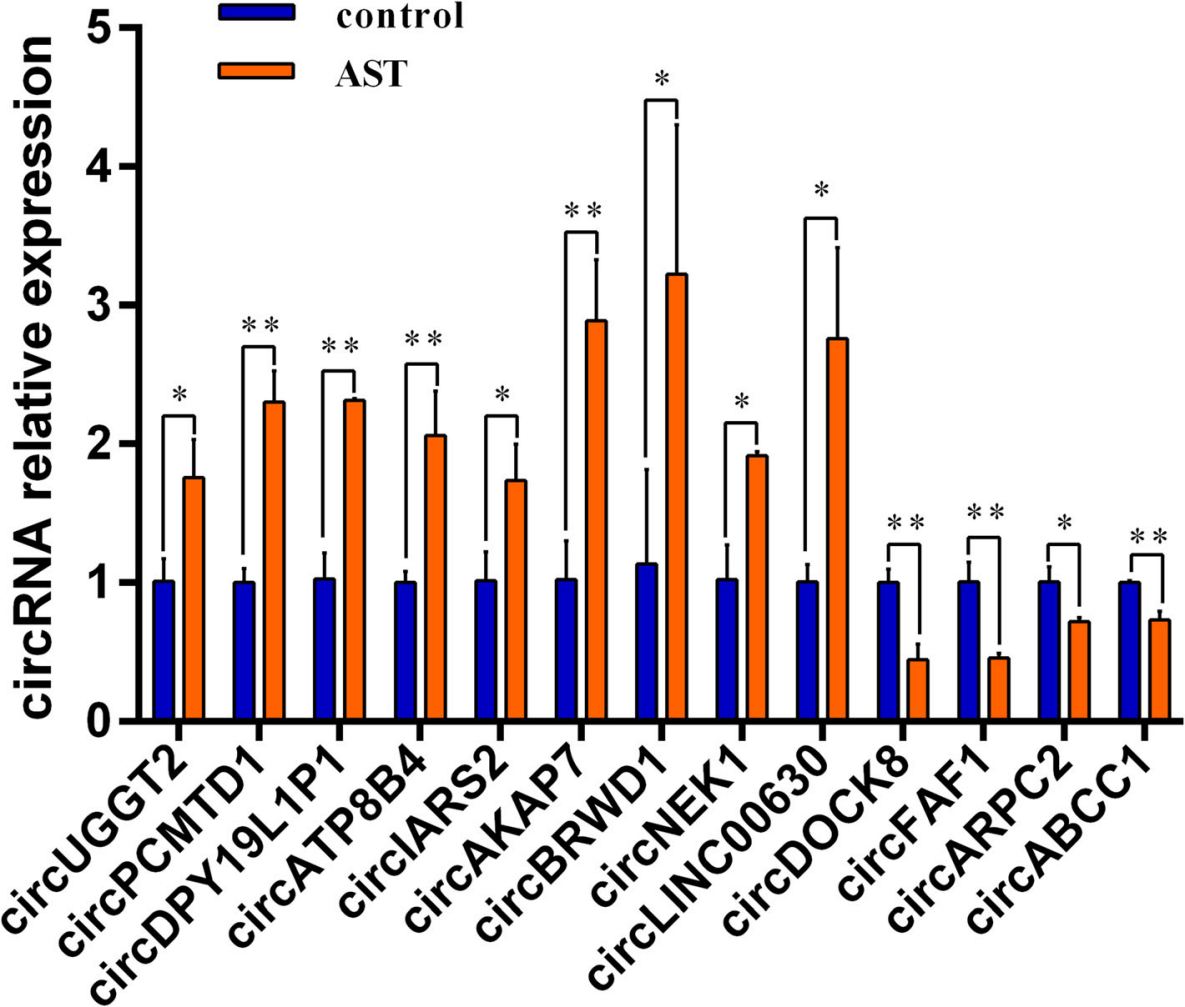
The 13 circRNAs are differentially expressed in the process of AST promoting cholesterol efflux from macrophages. The expression of the first nine circRNAs was significantly upregulated, and the expression of the next four circRNAs was significantly downregulated. These trends were consistent with RNA-seq results. Data were expressed as the mean ± SD and were analyzed by Student’s *t* test. *P < 0.05 vs. control. **P < 0.01 vs. control

Among the 7276 circRNAs with a difference of more than 2 fold change, we selected the top 25 significantly upregulated and the top 25 significantly downregulated circRNAs and verified their expression by qRT-PCR. We found that the expression of circUGGT2, circPCMTD1 (hsa_circ_0001801), circDPY19L1P1, circATP8B4, circIARS2, circAKAP7, circBRWD1, circNEK1, circLINC00630 were differentially upregulated, and the expression of circDOCK8, circFAF1, circARPC2 (hsa_circ_0058218), circABCC1 (hsa_circ_0000678) were differentially downregulated, which was consistent with RNA-seq results (Fig. 2). The results indicated that these circRNAs have potential in the process of AST promoting cholesterol efflux from macrophages.

### Analysis of differentially expressed circRNAs targeted miRNAs

**Fig. 3:**
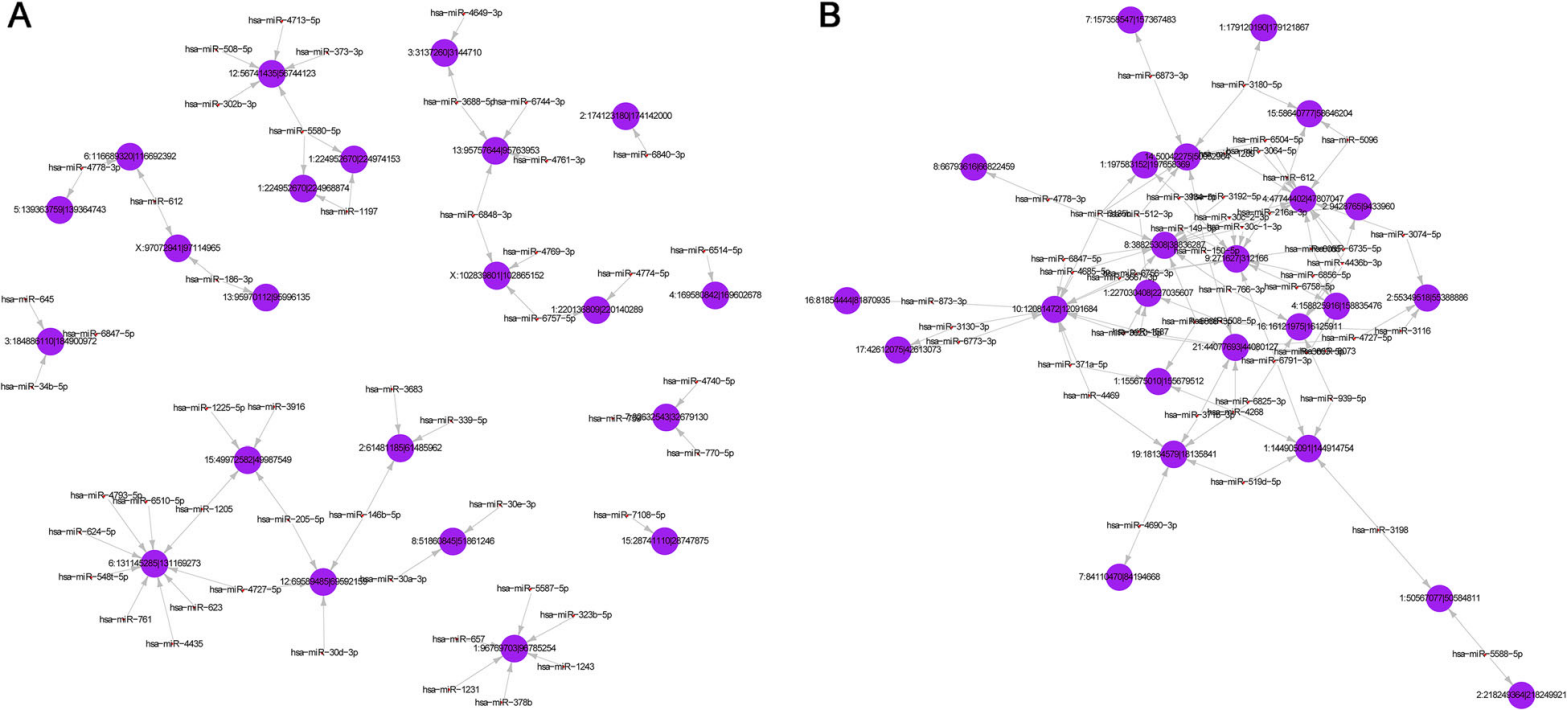
The circRNA targeted miRNA network. Top 50 putative target miRNAs of the upregulated circRNAs (**A**) and the downregulated circRNAs (**B**) based on the *P*-value sorting information. The purple nodes represent the circRNAs, the red nodes represent the targeted miRNAs, and each gray line means a possible interaction of one circRNA with its targeted miRNA

It has been shown that the MREs of circRNAs can bind matched miRNA, and thereby reduce miRNA-mediated post-transcriptional repression. To explore the potential functions of the circRNAs, we predicted the target miRNA by aligning with the MREs of these 25 differentially upregulated and 25 downregulated circRNAs using the miRanda software. We found that at least one miRNA with one differentially expressed circRNA was co-targeted, so we chose each of the top 50 putative target miRNAs based on the *P*-value sorting information. The results from the sequence analysis were visually compiled in Fig. 3.

### siRNA interference screened out the 3 most differentially expressed circRNAs

**Fig. 4:**
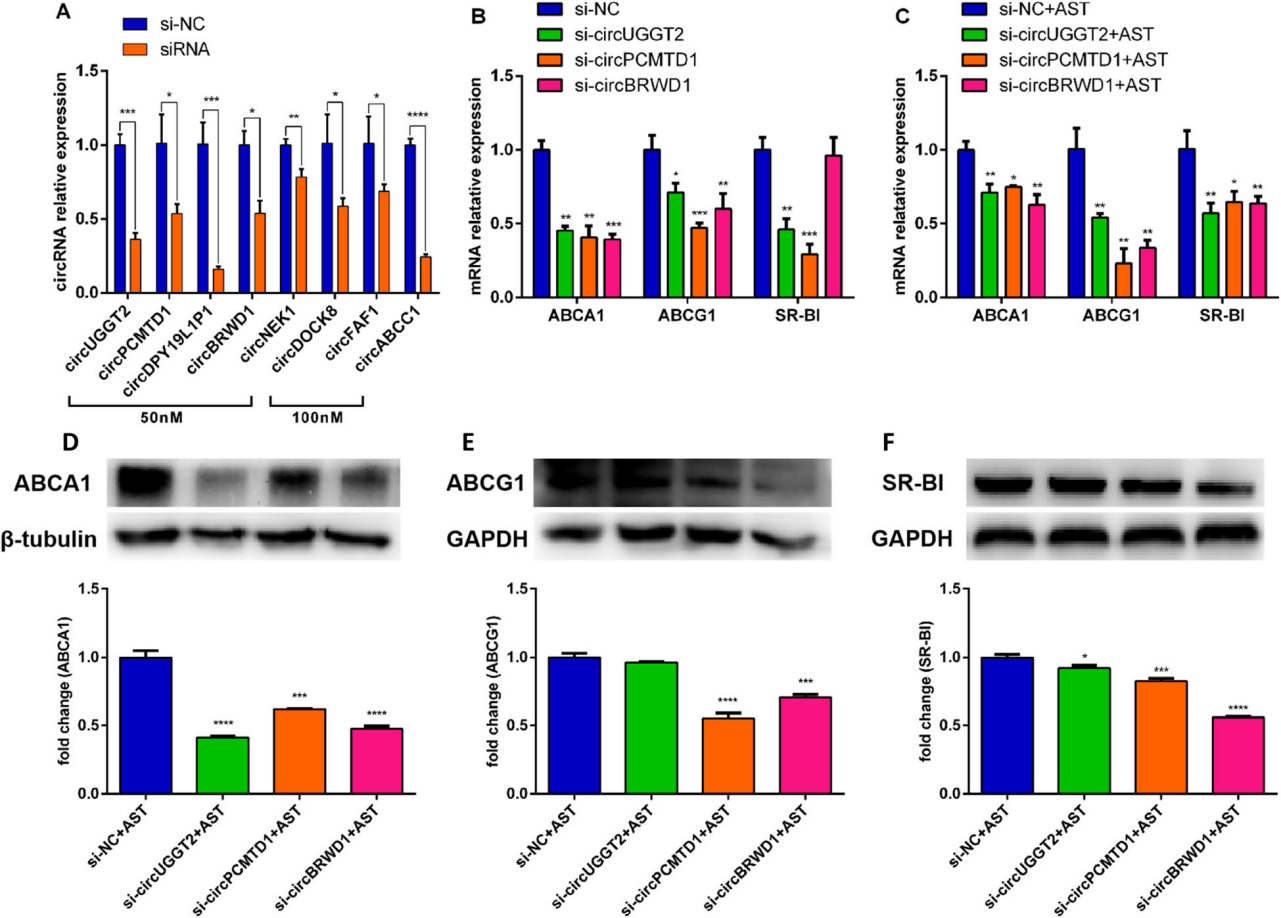
Effect of siRNA interference with the expression of circRNAs on target genes and proteins. (**A**) PCR to verify the interference effect of different concentrations of siRNA. (**B**) The effect of siRNA interference on ABCA1, ABCG1, and SR-BI target genes. (**C**) The effect of circRNAs interference on ABCA1/G1, and SR-BI genes in the AST intervention cell model. (**D**–**F**) The effect of circRNAs interference on ABCA1/G1, and SR-BI proteins in the AST intervention cell model. *P < 0.05 vs. control. **P < 0.01 vs. control. ***P < 0.001 vs. control. ****P < 0.0001 vs. control

The 13 circRNAs verified by qRT-PCR (9 upregulated and 4 downregulated) were designed to interfere with siRNA, and the appropriate concentration was explored to verify the interference effect of the circRNAs. The results showed that the siRNA concentration of circUGGT2, circPCMTD1, circDPY19L1P1, circBRWD1, and circNEK1 was better when the siRNA concentration of the 5 circRNAs was 50 nM, while the siRNA of circDOCK8, circFAF1, and circABCC1 was better at 100 nM (Fig. 4A). The effects of these 8 circRNA interferences on the mRNA and protein levels of ABCA1, ABCG1, and SR-BI were further tested. The final results found that the 3 target genes were significantly reduced after interference with circUGGT2, and circPCMTD1, while ABCA1 and ABCG1 were significantly reduced after interference with circBRWD1, but SR-BI was not obvious (Fig. 4B). At the same time, in the AST intervention cell model, compared with the negative control, the gene expression levels of ABCA1, ABCG1, and SR-BI were significantly reduced after interfering with the 3 circRNAs (Fig. 4C), and their protein levels were also reduced. (Fig. 4D–F).

### Targeted miRNAs related to 3 circRNAs

**Fig. 5:**
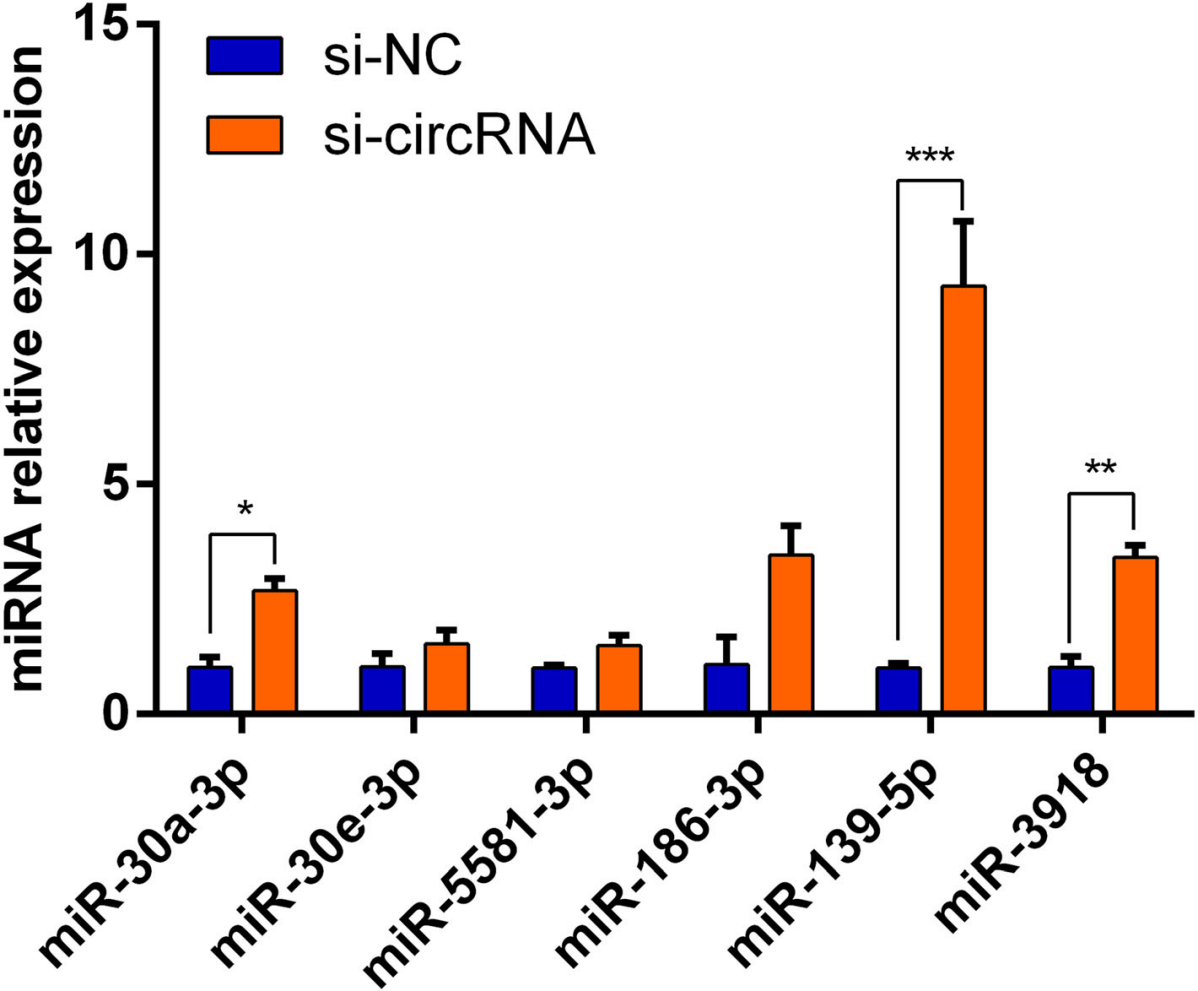
Effect of siRNA interference with the expression of circRNAs on target miRNAs. qRT-PCR verified that the expression of miRNAs were upregulated after siRNA interferes with circRNAs. *P < 0.05 vs. control. **P < 0.01 vs. control. ***P < 0.001 vs. control

Predicted by TargetScan, starBase, miRanda, and miRDB software, we got miRNAs related to the 3 target genes of ABCA1, ABCG1, and SR-BI. After intersecting the related target miRNAs of the 3 circRNAs predicted by the sequencing results, we got a total of 6 miRNAs. (Table 1). After siRNA interfered with circRNAs, qRT-PCR verified the expression of 6 miRNAs, and the results showed that all miRNAs were increased to different degrees. Among them, miR-30a-3p, miR-139-5p, and miR-3918 were significantly increased, indicating that circRNAs may play a role in AST’s promotion of cholesterol efflux by targeting miRNA (Fig. 5).

**Table 1:** Circular RNA and target gene predicted co-targeting miRNA

CircRNA	Targeted miRNA	Targeted gene
circUGGT2	hsa-miR-186-3p	ABCA1
circPCMTD1	hsa-miR-30a-3p	ABCA1
circPCMTD1	hsa-miR-30e-3p	ABCA1
circPCMTD1	hsa-miR-5581-3p	ABCG1
circBRWD1	hsa-miR-139-5p	ABCA1
circBRWD1	hsa-miR-3918	ABCG1/SR-BI

## Discussion

The excessive accumulation of LDL-C and other lipids in macrophages in the arterial intima will lead to the formation of foam cells, which is the main sign of early atherosclerotic lesions [4]. The oxidation of LDL (that is, the formation of ox-LDL) stimulates the infiltration of monocytes to induce endothelial cell apoptosis and the formation of foam cells to promote AS [23]. Therefore, removing excess cholesterol through the RCT pathway can inhibit anti-AS [24]. RCT is an important part of the mechanism of cholesterol homeostasis in the body, and cholesterol efflux is the first and most important step in this process [25, 26]. The most researched on the mechanism of cholesterol efflux is the active transport mediated by transporters ABCA1, ABCG1 and SR-BI [4, 8]. Studies have reported that AS can enhance apolipoprotein AI and HDL-mediated cholesterol excretion in RAW264.7 cells, promote the expression of ABCA1, ABCG1 and significantly reduce the expression of class A scavenger receptors and CD36 [15, 27].

Therefore, in this study, we first used THP-1 cells loaded with ox-LDL to form foam cells as an in vitro model of atherosclerosis to study the mechanism of circRNAs in the promotion of cholesterol efflux by AST. The results of this article show that AST can significantly promote the outflow of lipid cholesterol in THP-1 cells, and can increase the expression of ABCA1, ABCG1, and SR-BI at the mRNA and protein levels. Therefore, it can be explained that AST can promote the outflow of ox-LDL, reduce the formation of foam cells, and promote the RCT process.

As a closed molecule without a 5′cap or 3′poly (A) tail, circRNA has a stable structure and plays a role in various diseases such as AS [28]. Research on the mechanism of circRNAs in AS is mostly focused on the following: acting as a miRNA sponge to competitively bind to endogenous RNA, regulating gene expression at the transcription, post-transcriptional and translation levels, and regulating ribosomal RNA maturation [29–31]. In the present study, a cell model of AST promoting cholesterol efflux was constructed for high-throughput sequencing, and the differentially expressed circRNAs were analyzed, and finally detected 7276 circRNAs. To validate these RNA-seq results, qRT-PCR was performed. The top 50 circRNAs were selected for qRT-PCR from the circRNAs detected in the control and treat cells, among which 25 circRNAs were upregulated and 25 were downregulated (|log_2_ (fold change)| > 2). Of these, 13 exhibited a statistically significant difference that was consistent with that of the RNA-seq detection. Specifically, 9 circRNAs, including circUGGT2, circPCMTD1, circDPY19L1P1, circATP8B4, circIARS2, circAKAP7, circBRWD1, circNEK1, and circLINC00630, were significantly upregulated, while 4 circRNAs, including circDOCK8, circFAF1, circARPC2, and circABCC1, were significantly downregulated.

Most of the new circRNAs were found in these 13 circRNAs, and only 3 circRNAs have relevant information in the circBase database: circPCMTD1 (hsa_circ_0058218), circARPC2 (hsa_circ_0058218), and circABCC1 (hsa_circ_0000678). Research has found that circPCMTD1 can act as a miR-224-5p sponge to promote the progression of glioma and promote the progression of glioblastoma by regulating the miR-628-5p/HMGB3 axis [32, 33]. In addition, exosomes carrying circABCC1 can mediate the stemness and metastasis of colorectal cancer cells and can be used as a new diagnostic biomarker for colorectal cancer [34, 35]. Further interfering with the expression of circRNAs by siRNA, we finally get 3 circRNAs that may play a role by targeting miRNA in the promotion of cholesterol efflux by AST: circUGGT2, circPCMTD1, and circBRWD1. Their specific mechanisms need to be further studied.

GO and KEGG functional analysis evaluated the main biological functions of the circRNA-miRNA-mRNA network and found that circRNAs are mainly related to the metabolic process in the biological process, and the most enriched molecular functions include binding to RNA and protein. The first 25 upregulated and the first 25 downregulated circRNAs with the most obvious differential expression were further selected as targets for screening and verification, and the miRanda software was combined to screen the targeted miRNAs, and the first 50 were selected to construct a network diagram. The results showed that one circRNA can bind to multiple miRNAs, and one miRNA can also bind to multiple circRNAs.

Our bioinformatics analysis determined that the expression of a large number of circRNAs in the promotion of cholesterol efflux by AST was significantly changed, and we screened out 3 circRNAs that might play a role. However, although we have preliminarily predicted circRNA functions from our sequencing results, it would be premature to use these circRNAs as possible AS biomarkers or therapeutic targets. Because of acknowledged limitations of this work, such as the small number of samples and individual differences in RNA-seq data, the biological functions of circRNAs and their role in the process of AST promoting cholesterol efflux require further study.

## Conclusions

We have verified that circRNAs are differentially expressed in the promotion of cholesterol efflux by AST through RNA-seq analysis and qRT-PCR verification, and finally found that 3 circRNAs are significantly differentially expressed in the process. This lays the foundation for the study of the specific mechanism and provides new discoveries for circRNAs as a target and marker for the diagnosis and treatment of AS in the future.

## Material and methods

### Cell culture and foam cell induction

THP-1 cells were cultured in RPMI-1640 medium containing 10% fetal bovine serum, 100 U/ml penicillin, and 100 μg/ml streptomycin in a 37 °C, 5% CO_2_ incubator, and passage every 2–3 days. The cultured cells were added with PMA at a final concentration of 100 ng/ml and cultured for about 48 h. The adherence, irregular shape, and pseudopodia of the cells were observed under the microscope to indicate that they had differentiated into macrophages. After the induced macrophages were cultured, the final concentration of 50 μg/ml ox-LDL was added and cultured for 24 h to form foam cells.

### Oil red O staining

THP-1 cells were induced to differentiate with 100 ng/ml PMA for 48 h and then treated with 50 μg/ml ox-LDL and 50 μM AST for 48 h as the treatment group, compared with the control group treated with 50 μg/ml ox-LDL, the oil red O staining kit (Solarbio, China) was used to detect the effect of AST on the foam formation of macrophages. First, remove the cell culture medium, wash twice with PBS, fixed with ORO Fixative for 20–30 min; discard the fixative and wash twice with distilled water; then add 60% isopropanol to soak for 5 min; then discard, add freshly prepared ORO Stain, soak for 10–20 min; then discard and wash 2–5 times with water until there is no excess staining solution; finally add Mayer hematoxylin staining solution, counter-stain the nucleus for 1–2 min (can be omitted), discard the dye solution, and wash with water 2–5 times; put in ORO Buffer for 1 min, discard it, add distilled water to cover the cells, and observe under a microscope.

### qRT-PCR

THP-1 cells were induced to differentiate and treated with 50 μg/ml ox-LDL plus different concentrations of AST (0, 0.5, 5, 50 μM) for 48 h. The cells were collected and extracted by Trizol reagent (Invitrogen, USA) in accordance with the manufacturer’s protocol. RNA quantity was determined spectrophotometrically at optical density (OD)_260_ and OD_260_/OD_280_ = 1.8–2.1 using a NanoDrop spectrophotometer (Thermo Fisher Scientific, Waltham, MA, USA). The circRNAs were treated with Rnase R (Geneseed, Guangzhou, China) for reverse transcription. Take 1 μg total RNA to use the reverse transcription kit (Roche, Shanghai, China) to obtain cDNA. qRT-PCR was conducted using the SYBR-Green MasterMix (Roche, Shanghai, China) in the Real-time PCR System (Applied Biosystems, Waltham, MA, USA). The oligonucleotide sequences of the mRNAs and circRNAs primers are in Table 2. Each qRT-PCR reaction included 5 μl SYBR, 0.15 μl forward primer, 0.15 μl reverse primer, and 1 μl complementary DNA. The total volume was adjusted to 10 μl with double-distilled H_2_O. The following thermocycler parameters were used to generate the dissociation curve: 95 °C for 10 min; and 40 cycles of 95 °C for 15 s, 56 °C for 30 s. mRNA and circRNA expression were normalized using glyceraldehyde-3-phosphate dehydrogenase and 18s rRNA respectively. The relative quantification was calculated by the 2^−ΔΔCt^ method.

**Table 2:** List of mainly primer sequences of the qRT-PCR

Name	Primer sequences (5′→3′)
GAPDH	F: GGAGTCAACGGATTTGGTCGTATTG
	R: TCTCGCTCCTGGAAGATGGTGAT
ABCA1	F: GATCTGGAAGGCATGTGG
	R: CTGTTCCCAAAAGTGGTCA
ABCG1	F: CCGGGCAGAGAGGTAAA R: AGGGGTTGGATCAGAAGAG
SR-BI	F: GGTCCATCTACCCACCCA R: CAGCGTTGAGGAAGTGAGG
circUGGT2	F: TCAGCCAGGCGATGCTCGTC R: TCCCAAGATTGCGAAGGCCATTC
circPCMTD1	F: AGCAAGCCTTCAGAGCGATTGATC R: TGCAAGGTGCTGACAAGTGGATG
circDPY19L1P1	F: GGCCTGGTTACAGTGTTGTGCTTC R: GCTTTCGCGGAGAGGTGGTG
circATP8B4	F: GCAATACCTGCAGTGGCTCG R: GTGAGGGTACCCGTTTTGTCG
circIARS2	F: TGATGTCCTTCGCTGGTGGGTAG R: TGGCAGCATTGAGCACGGATG
circAKAP7	F: TCCTGTCCATTCCAATCACCA R: GGCCAGTCGCTCATCTTGTT
circBRWD1	F: TAGCAGCAGCTTTTGCAGGC R: TAACGCAGACAGCCTCCTGTA
circNEK1	F: GAGCTGGCTCGAACTTGCATA R: AAAGGACACACCCCAGAGCC
circLINC00630	F: GGCTGCCAAAGCTTCTACTCC R: AGCAGGTGTCATGTCCTACCA
circDOCK8	F: GGGACTTCACTGATGACGACTTGG R: TAGCCACTCACGGATGTAGGTCTG
circFAF1	F: CCAACGTGTTCTGCTCACAAATGC R: GAGTCCTTTGTCAGATCCCAAGCC
circARPC2	F: AACACCATCAACCTGATCCACACG R: CGGTTCAGCACCTTGAGGAAGTC
circABCC1	F: TCATTGCAGGTCACCACGTACTTG R: CTCTCCACGGCCACGATGTTG
18s rRNA	F: ACCTGGTTGATCCTGCCAG R: TCCAAGTAGGAGAGGAGCG

### Western blot analysis

The protein was extracted with RIPA lysate and protease inhibitor 100:1, and the BCA kit was used for protein quantification. After adding the loading buffer, the protein was boiled and stored at − 20 °C. The preserved protein samples were electrophoresed on a 6% SDS-PAGE gel at 80V 120 min; then 250 mA 150 min, electro-transported to the PVDF membrane; the skimmed milk powder was blocked for 1.5 h with rabbit anti-ABCA1 (1:1000, CST, USA), ABCG1 (1:500, Proteintech, Wuhan, China), SR-BI (1:500, Sangon Biotech, Shanghai, China) and GAPDH (1:1000, Sangon Biotech, Shanghai, China). Incubate overnight in a shaker at 4 °C, add horseradish peroxidase (HRP) labeled goat Anti-rabbit IgG (1:1000, Beyotime, Shanghai, China), using chemiluminescence western blot detection system to detect protein expression. Using GAPDH as an internal reference, the development results were analyzed for gray data results.

### RNA sample preparation for high-throughput sequencing

RNA samples collected from control and 50 μM AST treat group were used for sequencing analysis, and take at least 1 × 10^7^ cells from each group. Total RNA was extracted from each sample by Trizol reagent (Invitrogen, USA) according to the protocol of the manufacturer. For high-throughput sequencing, we synthesized the cDNA libraries of the circRNAs from each sample based on the Illumina standard protocols (Genergy Biotechnology, Shanghai, China). Briefly, the ribosomal RNAs were removed, and the linear RNAs were digested and removed with Rnase R, thus got the pure circRNAs. Transcriptome sequencing of RNA harvested from control and group cells was performed by Illumina HiSeq 2500. Primary sequencing data (raw reads) were subjected to quality control to filter out low-quality reads. The expression levels of mapped genes were calculated by the reads per kilobase transcriptome per million mapped reads method to normalize gene expression levels. Transcripts that had a fold change > 2 and *q*-value < 0.05 were considered to be significantly differentially expressed.

### Functional enrichment analysis

To demonstrate gene ontology or molecular pathway enrichment, we used the Database for Annotation, Visualization, and Integrated Discovery (DAVID) (http://david.abcc.ncifcrf.gov/) to determine the most functional annotation and classification of significant differentially expressed circRNAs target genes. GO encompasses three domains: biological process, cellular component, and molecular function, and provides extensive annotation of genes and gene products (http://www.geneontology.org). In addition, the KEGG pathway (http://www.genome.ad.jp/kegg/) was used to annotate and classify the functions of the target genes of the differently expressed circRNAs in the pathways. The *P*-values denote the significance of GO term enrichment or the significance of the KEGG pathway correlation (*P* < 0.05 was considered to be statistically significant).

### Differential expression analysis of circRNAs

The expression values of circRNAs in each sample were normalized using the Back Spliced Reads Per (BSRP) million mapped reads. BSRP is defined per million sequence number as the number of circRNA expression, of which the total number of aligned reads were used by normalized expression values, the number of sequences were used by the sequence number of circRNA back-splice regions, and the length of transcripts is the total length of the circRNA exon region. The sequencing data from the treatment group and the control group were analyzed and compared using DESEQ software. The log_2_ (fold change) was used as the standard to sort and select the top 25 upregulated and top 25 downregulated circRNAs (Table 3) with the most significant differential expression between the two groups.

**Table 3:** The significantly differentially expressed top 25 upregulated and top 25 downregulated circular RNAs

CircRNA ID	CircBase ID	Log_**2**_FC	Gene
6:116689320|116692392	—	6.768184	KPNA5
3:3137260|3144710	—	6.303781	TRNT1
13:95970112|95996135	—	5.930737	UGGT2
8:51860845|51861246	hsa_circ_0001801	5.61471	PCMTD1
7:32632543|32679130	—	5.554589	DPY19L1P1
12:69589485|69592159	hsa_circ_0000418	5.357552	CCT2
1:224952670|224974153	hsa_circ_0016600	5.285402	DNAH14
3:152455578|152456064	—	4.954196	MBNL1
15:49972582|49987549	—	4.954196	ATP8B4
15:28741110|28747875	—	4.857981	WHAMMP2
13:95757644|95763953	—	4.754888	DNAJC3
1:224952670|224968874	hsa_circ_0016599	4.754888	DNAH14
3:184886110|184900972	hsa_circ_0068367	4.643856	VPS8
1:220136809|220140289	—	4.523562	IARS2
1:92262863|92271652	hsa_circ_0013148	4.523562	GLMN
X:97072941|97114965	—	4.523562	DIAPH2
12:56741435|56744123	hsa_circ_0000409	4.523562	PRIM1
5:139363759|139364743	hsa_circ_0001538	4.523562	PAIP2
1:96769703|96785254	hsa_circ_0013252	4.392317	PTBP2
2:61481185|61485962	—	4.392317	XPO1
6:131145285|131169273	—	4.392317	AKAP7
21:39215237|39218660	—	4.392317	BRWD1
4:169580842|169602678	—	4.392317	NEK1
X:102839801|102865152	—	4.392317	LINC00630
2:174123180|174142000	hsa_circ_0057129	4.247928	OLA1
1:155675010|155679512	hsa_circ_0003608	-6.26679	YY1AP1
1:144905091|144914754	—	-4.9542	SRGAP2B
10:12081472|12091684	hsa_circ_0003074	-4.9542	DHTKD1
1:227030408|227035607	hsa_circ_0016738	-4.85798	CDC42BPA
9:271627|312166	—	-4.64386	DOCK8
21:44077693|44080127	hsa_circ_0003342	-4.52356	TRAPPC10
4:158825916|158835476	hsa_circ_0006432	-4.52356	FNIP2
7:157358547|157367483	hsa_circ_0083172	-4.52356	DNAJB6
17:42612075|42613073	hsa_circ_0006710	-4.52356	TUBG1
2:9428765|9433960	—	-4.52356	CPSF3
1:156324972|156334918	hsa_circ_0005131	-4.52356	CCT3
4:47744402|47807047	hsa_circ_0069643	-4.39232	CORIN
1:179120190|179121867	hsa_circ_0015430	-4.39232	ABL2
16:81854444|81870935	—	-4.39232	PLCG2
2:55349518|55388886	—	-4.39232	CCDC88A
8:66890865|66905335	—	-4.24793	MCMDC2
17:81613318|81629805	hsa_circ_0046209	-4.24793	NPLOC4
1:50567077|50584811	—	-4.24793	FAF1
8:38825308|38836287	—	-4.24793	TACC1
2:218249364|218249921	hsa_circ_0058218	-4.24793	ARPC2
1:197583152|197658369	—	-4.24793	DENND1B
8:66793616|66822459	—	-4.24793	SGK3, C8orf44-SGK3
16:16121975|16125911	hsa_circ_0000678	-4.24793	ABCC1
14:50042275|50052904	—	-4.24793	LINC01588
19:18134579|18135841	—	-4.24793	MAST3

### Target miRNA of circRNAs prediction

CircRNAs act as miRNA sponges and play a crucial role in the miRNA-mediated post-transcriptional gene regulation by binding to multiple miRNA recognition elements (MREs) and sequestering miRNAs. To investigate the functional annotation of identified circRNAs, we evaluated putative interactions between the miRNA sequences and the predicted circRNAs using the miRanda software. The top 50 putative target miRNAs based on the *P*-value sorting information were identified from the above analysis. A circRNA-miRNA network was generated to visualize the interactions.

### siRNA transfection

The siRNAs targeting circRNAs (sequences were listed in supplementary material: Table S1) were designed and synthesized by GeenPharma (Shanghai, China). Inoculate the cells the day before transfection so that the density of the cells during transfection is 30%–50%. A RFect transfection reagent (Baidai, Changzhou, China) was used to transfect siRNAs into cells in accordance with the manufacturer’s protocol. After 24 h of transfection, the medium was changed and added 50 μg/ml ox-LDL and 5 μM AST, and the cells were collected after 48 h of treatment.

### Statistical analysis

In the circRNA-seq, the circRNAs expressed values of each sample were calculated using the Back Spliced Reads Per million mapped reads (BSRP). The differentially expressed circRNAs were selected with a log_2_ (fold change) ≥ 1.0 and FDRs (false discovery rates) ≤ 0.05 with statistical significance. In the qRT-PCR analysis, expression levels were calculated by 2^−△ct^ and fold change was calculated by the 2^−△△ct^ method. The statistical analysis was analyzed using GraphPad Prism version 5.0 (GraphPad Software, USA). Each value is shown as the mean ± standard error of the mean (SEM). The data analysis was performed by Student’s *t* test and *P* < 0.05 was considered statistically significant.

## Supplementary Information

**Additional file 1: Supplementary Table S1.** siRNA sequences of the 13 circRNAs. **Supplementary Fig. S1.** CircRNAs differentially expression pattern of the treat and control groups. The significant differentially expressed circRNAs between the two groups were illustrated in the volcano plot (A) and the scatter plot (B). The volcano plot showed the fold changes and *P*-values of circRNAs. The green and red blots of the scatter plot mean the significant DE-circRNAs. The black blots mean the non-significant DE-circRNAs. In the heat map (C), the color scale reflects the log_2_ signal intensity and runs from blue (low intensity), to white (medium intensity), to red (strong intensity). Upregulated circRNAs are shown in red, and downregulated circRNAs are shown in blue. **Supplementary Fig. S2.** Significantly enriched GO histogram and dendrogram. GO analysis providing information concerning significantly enriched functions and the corresponding differentially expressed circRNAs covering 3 domains: biological process (BP), cellular component (CC) and molecular function (MF). According to the *P*-value ≤ 0.05 to filter the significant accumulation GO (A), the top 10 GOs are displayed under each GO category. The dendrogram (B) is CC, MF, BP from top to bottom, and the depth of the color indicates the degree of enrichment. The deeper the color, the higher the degree of enrichment. **Supplementary Fig. S3.** KEGG pathway histogram and scatter plot. (A) The histogram and (B) scatter plot showed the KEGG enriched analysis of circRNA-miRNA-mRNAs network of differentially expressed circRNAs. The top 20 significantly enriched pathway and their scores (negative logarithm of *P*-value) were listed as the x-axis and the y-axis, respectively.

## Data Availability

All data supporting the conclusions of this article are included within the articles and the supplementary materials.

## Abbreviations

circRNA: Circular RNA
AS: Atherosclerosis
LDL-C: Low-density lipoprotein cholesterol
HDL-C: High-density lipoprotein cholesterol
RCT: Reverse cholesterol transport
ABCA1: ATP-binding cassette transporter A1
ABCG1: ATP-binding cassette transporter G1
SR-BI: Scavenger receptor class B type I
AST: Astaxanthin
THP-1: Human acute monocytic leukemia cell line
ox-LDL: Oxidized low-density lipoprotein
PMA: Phorbol-12-myristate-13-acetate
qRT-PCR: Real-time quantitative PCR
GO: Gene ontology
KEGG: Kyoto encyclopedia of genes and genomes
RT: Reverse transcription
SDS: Sodium dodecyl sulfate
PBS: Phosphate-buffered saline
BSRP: Back spliced reads per
MREs: MiRNA recognition elements
Rnase R: Ribonuclease R

## References

[1] Wang C, Niimi M, Watanabe T, Wang Y, Liang J, Fan J (2018). Treatment of atherosclerosis by traditional Chinese medicine: questions and quandaries. Atherosclerosis..

[2] Kobiyama K, Ley K (2018). Atherosclerosis. Circ Res..

[3] Wang HH, Garruti G, Liu M, Portincasa P, Wang DQ (2017). Cholesterol and lipoprotein metabolism and atherosclerosis: recent advances in reverse cholesterol transport. Ann Hepatol..

[4] Yu XH, Fu YC, Zhang DW, Yin K, Tang CK (2013). Foam cells in atherosclerosis. Clin Chim Acta..

[5] Tosheska Trajkovska K, Topuzovska S (2017). High-density lipoprotein metabolism and reverse cholesterol transport: strategies for raising HDL cholesterol. Anatol J Cardiol..

[6] Tang SL, Zhao ZW, Liu SM, Wang G, Yu XH, Zou J, Wang SQ, Dai XY, Fu MG, Zheng XL, Zhang DW, Fu H, Tang CK (2019). Pregnancy-associated plasma protein-A accelerates atherosclerosis by regulating reverse cholesterol transport and inflammation. Circ J..

[7] Ouimet M, Barrett TJ, Fisher EA (2019). HDL and reverse cholesterol transport. Circ Res..

[8] Chistiakov DA, Melnichenko AA, Myasoedova VA, Grechko AV, Orekhov AN (2017). Mechanisms of foam cell formation in atherosclerosis. J Mol Med..

[9] Sharma B, Agnihotri N (2019). Role of cholesterol homeostasis and its efflux pathways in cancer progression. J Steroid Biochem Mol Biol..

[10] Phillips MC (2014). Molecular mechanisms of cellular cholesterol efflux. J Biol Chem..

[11] Li J, Xia Y, Liu T, Wang J, Dai W, Wang F, Zheng Y, Chen K, Li S, Abudumijiti H, Zhou Z, Wang J, Lu W, Zhu R, Yang J, Zhang H, Yin Q, Wang C, Zhou Y, Lu J, Zhou Y, Guo C (2015). Protective effects of astaxanthin on ConA-induced autoimmune hepatitis by the JNK/p-JNK pathway-mediated inhibition of autophagy and apoptosis. PLoS One..

[12] Kumar R, Salwe KJ, Kumarappan M (2017). Evaluation of antioxidant, hypolipidemic, and antiatherogenic property of lycopene and astaxanthin in atherosclerosis-induced rats. Pharmacognosy Res..

[13] Gasmi A, Mujawdiya PK, Shanaida M, Ongenae A, Lysiuk R, Doşa MD, Tsal O, Piscopo S, Chirumbolo S, Bjørklund G (2020). Calanus oil in the treatment of obesity-related low-grade inflammation, insulin resistance, and atherosclerosis. Appl Microbiol Biotechnol..

[14] Kishimoto Y, Yoshida H, Kondo K (2016). Potential anti-atherosclerotic properties of astaxanthin. Mar Drugs..

[15] Iizuka M, Ayaori M, Uto-Kondo H, Yakushiji E, Takiguchi S, Nakaya K (2012). Astaxanthin enhances ATP-binding cassette transporter A1/G1 expressions and cholesterol efflux from macrophages. J Nutr Sci Vitaminol..

[16] López-Jiménez E, Rojas AM, Andrés-León E (2018). RNA sequencing and prediction tools for circular RNAs analysis. Adv Exp Med Biol..

[17] Geng X, Jia Y, Zhang Y, Shi L, Li Q, Zang A, Wang H (2020). Circular RNA: biogenesis, degradation, functions and potential roles in mediating resistance to anticarcinogens. Epigenomics..

[18] Hsiao KY, Sun HS, Tsai SJ (2017). Circular RNA-new member of noncoding RNA with novel functions. Exp Biol Med..

[19] Wang L, Zheng Z, Feng X, Zang X, Ding W, Wu F, Zhao Q (2019). CircRNA/lncRNA-miRNA-mRNA network in oxidized, low-density, lipoprotein-induced foam cells. DNA Cell Biol..

[20] Mao YY, Wang JQ, Guo XX, Bi Y, Wang CX (2018). Circ-SATB2 upregulates STIM1 expression and regulates vascular smooth muscle cell proliferation and differentiation through miR-939. Biochem Biophys Res Commun..

[21] Yang L, Yang F, Zhao H, Wang M, Zhang Y (2019). Circular RNA circCHFR facilitates the proliferation and migration of vascular smooth muscle via miR-370/FOXO1/Cyclin D1 pathway. Mol Ther Nucleic Acids..

[22] Shen L, Hu Y, Lou J, Yin S, Wang W, Wang Y, Xia Y, Wu W (2019). CircRNA-0044073 is upregulated in atherosclerosis and increases the proliferation and invasion of cells by targeting miR-107. Mol Med Rep..

[23] Mertens A, Holvoet P (2001). Oxidized LDL and HDL: antagonists in atherothrombosis. Faseb J..

[24] Yu XH, Zhang DW, Zheng XL, Tang CK (2019). Cholesterol transport system: an integrated cholesterol transport model involved in atherosclerosis. Prog Lipid Res..

[25] Favari E, Chroni A, Tietge UJ, Zanotti I, Escolà-Gil JC, Bernini F (2015). Cholesterol efflux and reverse cholesterol transport. Handb Exp Pharmacol..

[26] Getz GS, Reardon CA (2018). Apoprotein E and reverse cholesterol transport. Int J Mol Sci..

[27] Kishimoto Y, Tani M, Uto-Kondo H, Iizuka M, Saita E, Sone H, Kurata H, Kondo K (2010). Astaxanthin suppresses scavenger receptor expression and matrix metalloproteinase activity in macrophages. Eur J Nutr..

[28] Meng S, Zhou H, Feng Z, Xu Z, Tang Y, Wu M (2019). Epigenetics in neurodevelopment: emerging role of circular RNA. Front Cell Neurosci..

[29] Holdt LM, Stahringer A, Sass K, Pichler G, Kulak NA, Wilfert W, Kohlmaier A, Herbst A, Northoff BH, Nicolaou A, Gäbel G, Beutner F, Scholz M, Thiery J, Musunuru K, Krohn K, Mann M, Teupser D (2016). Circular non-coding RNA ANRIL modulates ribosomal RNA maturation and atherosclerosis in humans. Nat Commun..

[30] Wei MY, Lv RR, Teng Z (2020). Circular RNA circHIPK3 as a novel circRNA regulator of autophagy and endothelial cell dysfunction in atherosclerosis. Eur Rev Med Pharmacol Sci..

[31] Zeng Z, Xia L, Fan S, Zheng J, Qin J, Fan X, Liu Y, Tao J, Liu Y, Li K, Ling Z, Bu Y, Martin KA, Hwa J, Liu R, Tang WH (2021). Circular RNA circMAP3K5 acts as a microRNA-22-3p sponge to promote resolution of intimal hyperplasia via TET2-mediated smooth muscle cell differentiation. Circulation..

[32] Zheng SQ, Qi Y, Wu J, Zhou FL, Yu H, Li L, Yu B, Chen XF, Zhang W (2019). CircPCMTD1 acts as the sponge of miR-224-5p to promote glioma progression. Front Oncol..

[33] Chen WL, Jiang L, Wang JS (2019). Circ-0001801 contributes to cell proliferation, migration, invasion and epithelial to mesenchymal transition (EMT) in glioblastoma by regulating miR-628-5p/HMGB3 axis. Eur Rev Med Pharmacol Sci..

[34] Zhao H, Chen S, Fu Q (2020). Exosomes from CD133^+^ cells carrying circ-ABCC1 mediate cell stemness and metastasis in colorectal cancer. J Cell Biochem..

[35] Lin J, Cai D, Li W, Yu T, Mao H, Jiang S, Xiao B (2019). Plasma circular RNA panel acts as a novel diagnostic biomarker for colorectal cancer. Clin Biochem..

