# Supplementary material for: Expression of the circular RNAs in astaxanthin promotes cholesterol efflux from THP-1 cells based on RNA-seq
Source: Genes Nutr. 2021 Aug 28;16:13. doi: 10.1186/s12263-021-00693-5 (PMC8403398; doi:10.1186/s12263-021-00693-5)
Supplement: Supplementary file 1 — Additional file 1: Supplementary Table S1. siRNA sequences of the 13 circRNAs. Supplementary Fig. S1. CircRNAs differentially expression pattern of the treat and control groups. The significant differentially expressed circRNAs between the two groups were illustrated in the volcano plot (A) and the scatter plot (B). The volcano plot showed the fold changes and P-values of circRNAs. The green and red blots of the scatter plot mean the significant DE-circRNAs. The black blots mean the non-significant DE-circRNAs. In the heat map (C), the color scale reflects the log2 signal intensity and runs from blue (low intensity), to white (medium intensity), to red (strong intensity). Upregulated circRNAs are shown in red, and downregulated circRNAs are shown in blue. Supplementary Fig. S2. Significantly enriched GO histogram and dendrogram. GO analysis providing information concerning significantly enriched functions and the corresponding differentially expressed circRNAs covering 3 domains: biological process (BP), cellular component (CC) and molecular function (MF). According to the P-value ≤ 0.05 to filter the significant accumulation GO (A), the top 10 GOs are displayed under each GO category. The dendrogram (B) is CC, MF, BP from top to bottom, and the depth of the color indicates the degree of enrichment. The deeper the color, the higher the degree of enrichment. Supplementary Fig. S3. KEGG pathway histogram and scatter plot. (A) The histogram and (B) scatter plot showed the KEGG enriched analysis of circRNA-miRNA-mRNAs network of differentially expressed circRNAs. The top 20 significantly enriched pathway and their scores (negative logarithm of P-value) were listed as the x-axis and the y-axis, respectively. [file 12263_2021_693_MOESM1_ESM.docx]

**Supplementary materials**


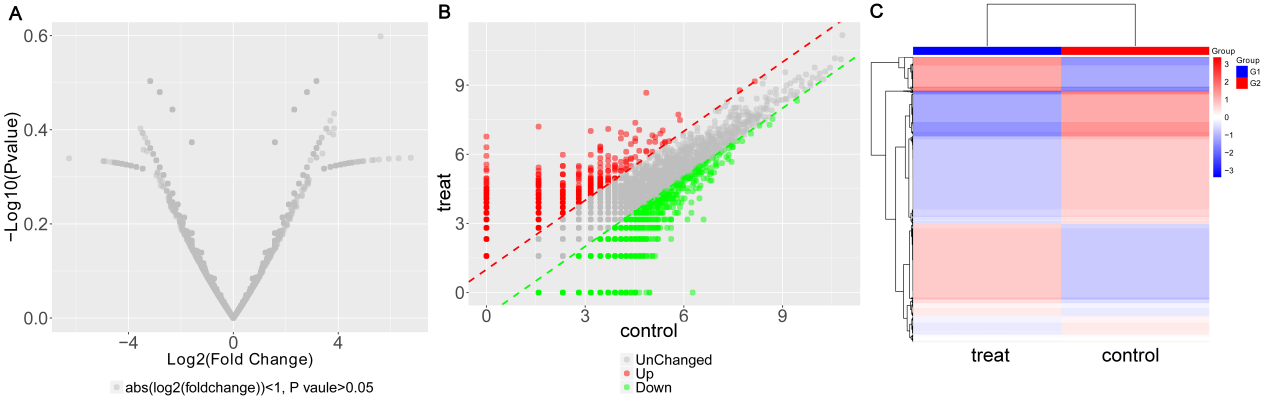


**Supplementary Fig. S1.** CircRNAs differentially expression pattern of the treat and control groups. The significant differentially expressed circRNAs between the two groups were illustrated in the Volcano plot (A) and the Scatter plot (B). The volcano plot showed the fold changes and p-values of circRNAs. The green and red blots of the scatter plot mean the significant DE-circRNAs. The black blots mean the non-significant DE-circRNAs. In the heat map (C), the color scale reflects the log_2_ signal intensity and runs from blue (low intensity), to white (medium intensity), to red (strong intensity). Up-regulated circRNAs are shown in red, and down-regulated circRNAs are shown in blue.


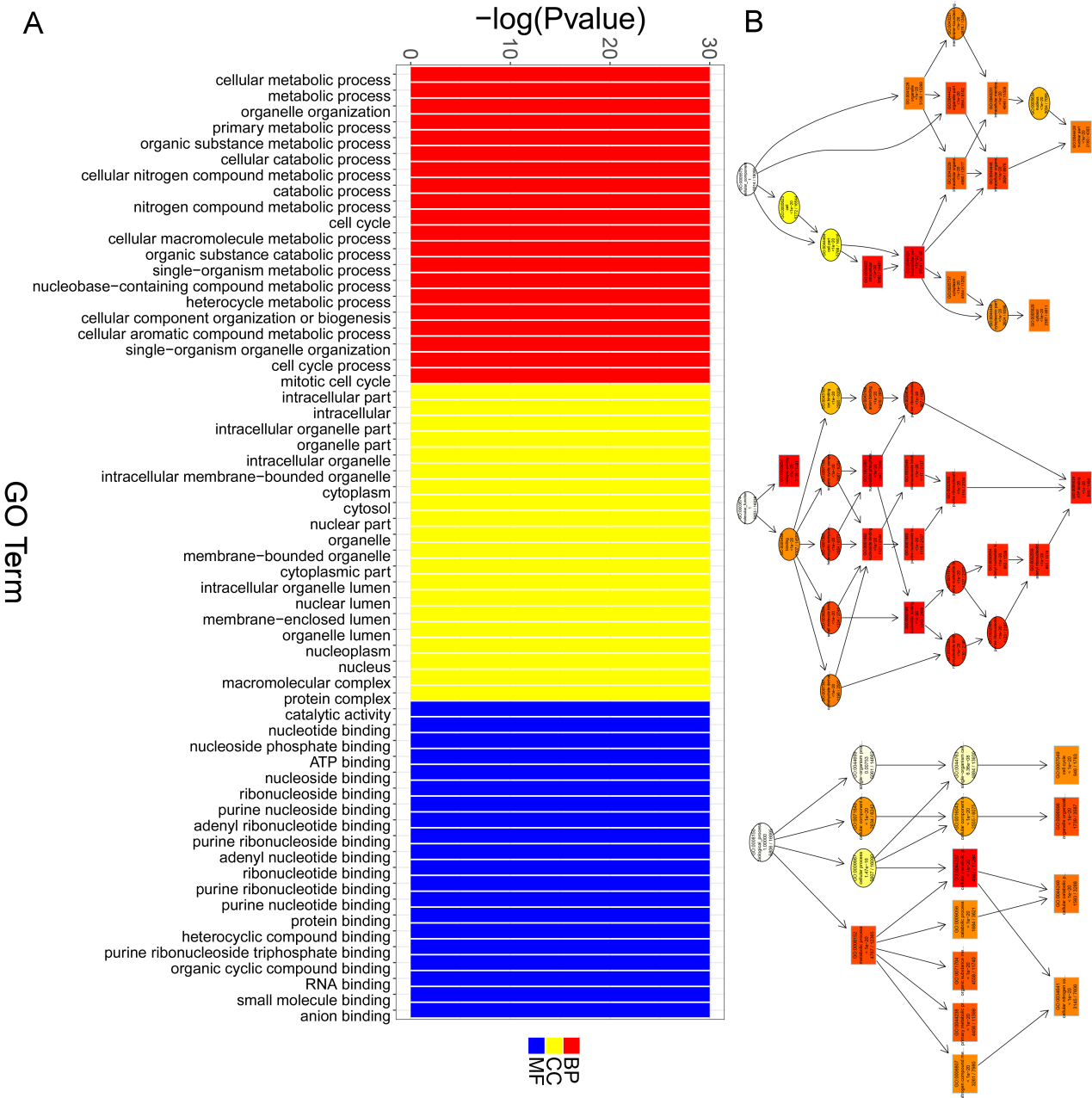


**Supplementary Fig. S2.** Significantly enriched GO histogram and dendrogram. GO analysis providing information concerning significantly enriched functions and the corresponding differentially expressed circRNAs covering three domains: biological process (BP), cellular component (CC) and molecular function (MF). According to the *P*-value ≤ 0.05 to filter the significant accumulation GO (A), the top 10 GOs are displayed under each GO category. The dendrogram (B) is CC, MF, BP from top to bottom, and the depth of the color indicates the degree of enrichment. The deeper the color, the higher the degree of enrichment.


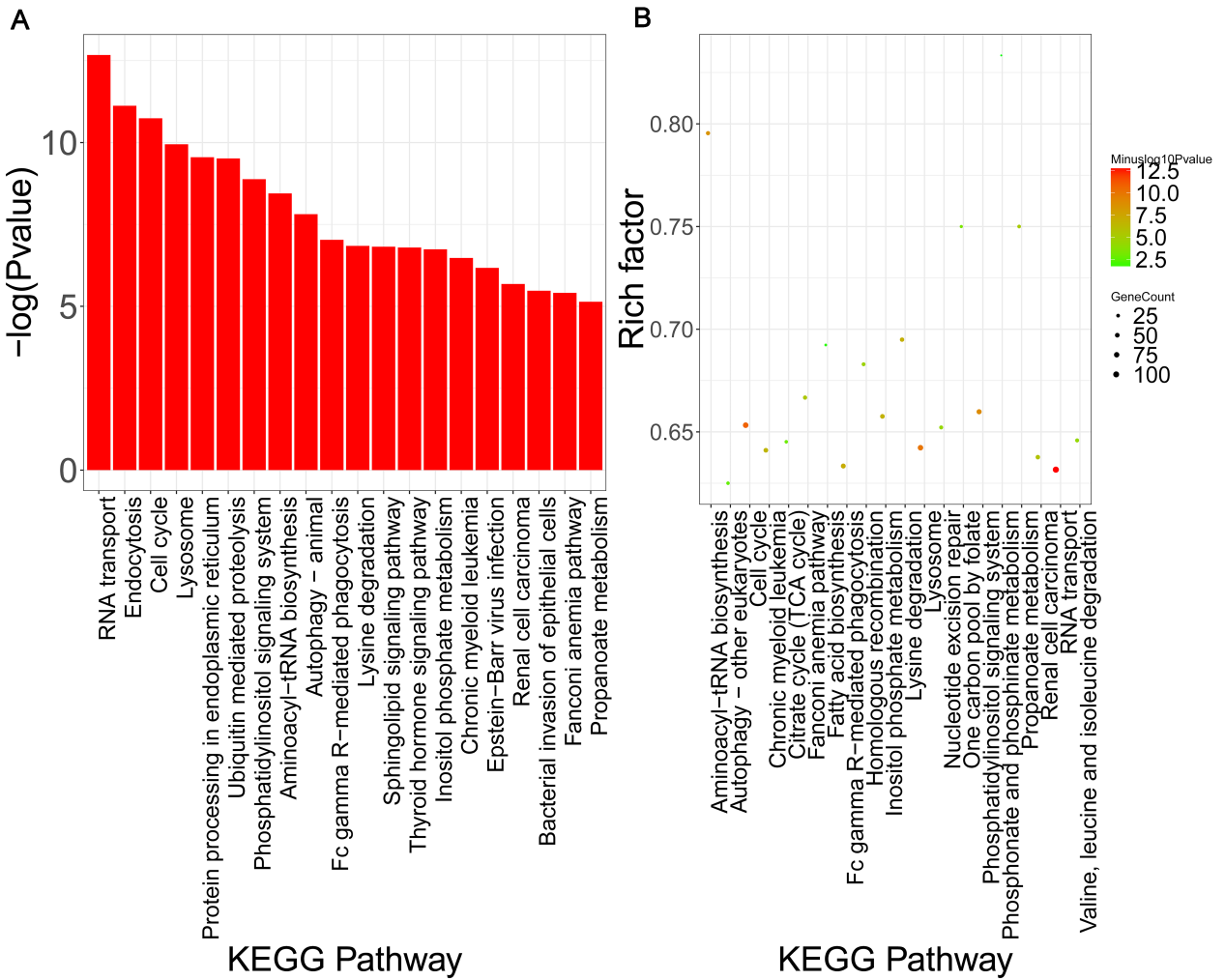


**Supplementary Fig. S3.** KEGG pathway histogram and scatter plot. (A) The histogram and (B) scatter plot shows the KEGG enriched analysis of circRNA-miRNA-mRNAs network of differentially expressed circRNA. The top 20 significantly enriched pathway and their scores (negative logarithm of *P* value) were listed as the x-axis and the y-axis, respectively.

**Supplementary Table S1.** siRNA sequences of the 13 circRNA

| circRNA | sense | antisense |
| --- | --- | --- |
| circUGGT2 | CAUUCUUCUAUAAUGCUGUTT | ACAGCAUUAUAGAAGAAUGTT |
| circPCMTD1 | GGGCUUAAUUUUAGGAUUATT | UAAUCCUAAAAUUAAGCCCTT |
| circDPY19L1P1 | AUAUACACAUGGGACUGUATT | UACAGUCCCAUGUGUAUAUTT |
| circATP8B4 | GGGAGAAUCUAUGGGAUUGTT | CAAUCCCAUAGAUUCUCCCTT |
| circIARS2 | CAAAGAUAGGACAGUGAUUTT | AAUCACUGUCCUAUCUUUGTT |
| circAKAP7 | GGAGAUAGCAGGAGGAAUUTT | AAUUCCUCCUGCUAUCUCCTT |
| circBRWD1 | AAUUUAUCAAUUGAUCCACTT | GUGGAUCAAUUGAUAAAUUTT |
| circNEK1 | CAGCCUAUACCAGUUUUUUTT | AAAAAACUGGUAUAGGCUGTT |
| circLINC00630 | GUUUAUAUUGAUAUUGGAATT | UUCCAAUAUCAAUAUAAACTT |
| circDOCK8 | CAUCAGUGGACGAGGUAUUTT | AAUACCUCGUCCACUGAUGTT |
| circFAF1 | GCAAGCAAAUCACCGAUGUTT | ACAUCGGUGAUUUGCUUGCTT |
| circARPC2 | CAAUCACUGCUGUUCCCUCTT | GAGGGAACAGCAGUGAUUGTT |
| circABCC1 | GACUGAGAAGGAGAGGUUCTT | GAACCUCUCCUUCUCAGUCTT |
| Negative control | UUCUCCGAACGUGUCACGUTT | ACGUGACACGUUCGGAGAATT |
